# Utility of artificial intelligence for the diagnosis, prognosis, and management of central serous chorioretinopathy: a narrative review

**DOI:** 10.1186/s40942-026-00867-6

**Published:** 2026-05-28

**Authors:** José Ignacio Fernández-Vigo, Alicia Valverde-Megías, Bárbara Burgos-Blasco, José Joaquim de Moura Ramos, Fernando Ly-Yang

**Affiliations:** 1 Department of Ophthalmology, Hospital Clínico San Carlos, San Carlos Health Research Institute (IdISSC), Profesor Martín Lagos s/n, Madrid, 28040 Spain; 2 Department of Immunology, Ophthalmology and ENT, Faculty of Medicine, Complutense University of Madrid, Madrid, Spain; 3 Centro Internacional de Oftalmología Avanzada, Madrid, Spain; 4 VARPA Research Group, Instituto de Investigación Biomédica de A Coruña (INIBIC), Universidad da Coruña, A Coruña, Spain; 5 Department of Ophthalmology, Birmingham and Midland Eye Centre, Sandwell and West Birmingham Hospital, NHS Trust, Birmingham, UK

**Keywords:** Central serous chorioretinopathy, Pachychoroid disease, Artificial intelligence, Machine learning, Biomarkers, Treatment strategies

## Abstract

Central serous chorioretinopathy (CSC) represents a significant cause of visual impairment, particularly in working-age individuals. Despite advances in multimodal imaging and evidence supporting photodynamic therapy (PDT) as the mainstay of chronic CSC, current clinical workflows are still affected by variability in image interpretation, manual quantification, and individualized treatment selection. This narrative review investigates the utility of Artificial Intelligence (AI) in improving the diagnosis, prognosis, and management of CSC. AI-based image analysis, including Optical Coherence Tomography (OCT), Optical Coherence Tomography Angiography (OCTA), and Fundus Fluorescein Angiography (FFA), has demonstrated high diagnostic accuracy across selected datasets and the ability to identify relevant biomarkers, thereby improving efficiency and consistency. Machine learning models demonstrate promising predictive power for subretinal fluid absorption, visual acuity outcomes, and disease recurrence, and identify critical prognostic factors. While emerging, AI-guided treatment strategies hold promise for personalized therapy, particularly for optimizing PDT, laser-based interventions, and follow-up strategies. The integration of AI into clinical decision-making workflows may elevate diagnostic capabilities, especially for non-specialists, and reduce clinical workload. However, the widespread implementation of AI in CSC faces notable challenges, including dataset bias, limited external validation, insufficient representation of differential diagnoses, regulatory complexities, and ethical considerations pertaining to transparency and equitable access. Future directions emphasize integrating multimodal data, fostering global collaborative efforts, and developing robust, generalizable AI models to fully realize AI’s potential to enhance patient care and optimize healthcare delivery for CSC.

## Introduction

Central serous chorioretinopathy (CSC) is an idiopathic retinochoroidal disease characterized by subretinal fluid (SRF) accumulation beneath the neurosensory retina, typically involving the central macula due to choroidal leakage through disruptions in the retinal pigment epithelium (RPE) [1, 2]. This condition is a significant cause of vision loss, ranking as the fourth most common non-surgical retinopathy worldwide, following age-related macular degeneration (AMD), diabetic retinopathy (DR), and retinal vein occlusion (RVO) [3, 4]. It primarily affects young men of working age, typically between 20 and 50 years, with an estimated incidence of approximately 10 per 100,000 in men and a male-to-female ratio ranging from 3:1 to 5:1 [5, 6]. 

The precise pathophysiology of CSC remains incompletely understood; however, current theories point to choroidal abnormalities, including increased thickness, hyperpermeability, and choroidal vessel congestion, as well as RPE dysfunction [7–9]. Recent evidence suggests that CSC may be part of the pachychoroid spectrum within venous overload choroidopathy, characterized by congestion of choroidal vessels and impaired venous outflow [7, 10]. The disease is often linked to endogenous or exogenous corticosteroid use and psychological factors, such as anxiety and Type A personality traits, which can trigger or worsen the condition [11, 12]. Clinically, patients with CSC typically present with central vision loss or distortion, central scotoma, metamorphopsia, dyschromatopsia, micropsia, and reduced contrast sensitivity [2, 13]. 

Acute CSC, usually defined by spontaneous resolution of SRF within 3 to 6 months, resolves in more than 80% of cases with good visual outcomes [3, 14]. Chronic CSC, characterized by persistent SRF lasting more than 6 months, can cause permanent RPE damage, outer retinal atrophy, and irreversible vision loss [3, 15]. Recurrence remains a major concern, with 20% to 30% of patients experiencing persistent or recurrent disease [6, 16]. The potential for lasting visual impairment in a demographically important, economically active population underscores the long-term societal and individual impact of this condition.

CSC diagnosis traditionally relies on clinical history, ophthalmoscopic examination, and multimodal imaging, including optical coherence tomography (OCT) and OCT angiography, fundus autofluorescence (FAF), fundus fluorescein angiography (FFA), and indocyanine green angiography (ICGA) [17, 18]. OCT is considered the most crucial diagnostic and decision-making tool because of its non-invasive nature, speed, and ability to provide high-resolution, cross-sectional, and three-dimensional images of retinal structures [17, 19]. Despite these advanced imaging techniques, current diagnostic and management approaches face several limitations. Manual assessment of OCT-generated volumetric data, including manual SRF segmentation, is feasible and has been successfully used in CSC research; however, it is time-consuming, may vary between graders, and is not always practical for routine longitudinal assessment [20]. Traditional diagnostic methods also rely on physician expertise and adequate clinical context, which can lead to variability, particularly in atypical presentations or in diseases with overlapping SRF. In addition, although PDT is well established as a mainstay of treatment for chronic CSC and is supported by multiple clinical studies and recent evidence-based recommendations [3, 21], treatment selection, timing, retreatment decisions, and standardized phenotyping remain areas in which objective, reproducible tools may add value. This combination of CSC’s potential for permanent visual impairment and the limitations of traditional, human-centric diagnostic and management processes creates a need for more efficient and scalable decision-support solutions.

Artificial intelligence (AI) is rapidly transforming ophthalmology by improving diagnostic accuracy, optimizing treatment strategies, streamlining clinical workflows, and enhancing patient outcomes [22–24]. Its ability to automate routine tasks reduces clinician workload and facilitates large-scale screening of prevalent retinal diseases [25, 26]. Ophthalmology is particularly well-suited for AI development due to its reliance on digital imaging and quantifiable parameters such as visual acuity and foveal thickness [22, 27]. This data-rich environment—especially in conditions like CSC, where multimodal imaging generates comprehensive, multidimensional datasets—provides an ideal foundation for advanced deep learning models. By integrating multimodal data, AI systems can detect complex patterns, improve disease subtyping, predict treatment response, and develop more robust diagnostic and prognostic models, potentially surpassing single-modality analysis or isolated human interpretation [22, 28, 29]. 

This narrative review aims to provide a comprehensive analysis of AI’s utility in CSC. It will cover AI’s applications in diagnosis, prognosis, and management, including its role in monitoring disease progression, integration into clinical decision-making processes, comparative analysis with traditional methods, and a critical review of related challenges, limitations, and ethical issues.

## Methods

The utility of AI in CSC was investigated through a narrative review of the scientific literature. The overarching research question, “Utility of Artificial Intelligence in the Diagnosis, Prognosis, and Management of Central Serous Chorioretinopathy,” was decomposed into detailed subtopics to ensure comprehensive coverage of the field.

A comprehensive search was conducted across medical and scientific databases to identify relevant studies, including PubMed/MEDLINE, ClinicalTrials.gov, Google Scholar, and the Cochrane Library. Studies published up to April 2026 were considered, without a lower date restriction. Additionally, relevant Food and Drug Administration (FDA) and European Medicines Agency (EMA) databases were queried to identify AI medical devices and software that have received regulatory approval. Only peer-reviewed and authoritative sources were included in the analysis.

A hierarchical prioritization was applied to the identified literature. Randomized controlled trials (RCTs), particularly those evaluating AI interventions, were given the highest priority. Meta-analyses and systematic reviews focusing on AI in ophthalmology were also highly valued. Subsequently, prospective and retrospective cohort studies evaluating AI models, validation studies of AI algorithms in CSC, and real-world evidence of AI deployment in CSC clinical settings were prioritized. This process identified areas of consensus, ongoing debate, and data scarcity, providing a balanced and nuanced overview of the current state of AI in CSC.

When available, key performance indicators were extracted to quantify the effectiveness of AI models. For diagnostic applications, metrics such as accuracy, sensitivity, specificity, and area under the receiver operating characteristic curve (AUC) were prioritized for CSC detection and subtyping. In the context of prognosis and recurrence prediction, predictive performance metrics, including R-squared, root-mean-square error (RMSE), and concordance index, were reported. The impact of AI on clinical workflow efficiency and diagnostic speed for CSC was also considered, along with reported false-positive and false-negative rates and their clinical implications. Comparative performance metrics of different AI architectures or algorithms were also extracted to evaluate their relative strengths.

Inherent limitations of the reviewed studies were acknowledged throughout the analysis. These included common challenges in AI model development, such as small sample sizes or single-center biases, which can restrict the generalizability of findings. The lack of external validation, a critical step for assessing real-world applicability, was also noted as a recurring limitation. Data heterogeneity and quality issues in training datasets were considered, as they can impact model robustness. Furthermore, the “black-box” nature of some AI models and the interpretability challenges they pose were recognized.

Compliance with established reporting guidelines, such as CONSORT-AI and STARD-AI, was evaluated to assess the transparency and reproducibility of AI studies [30, 31]. Finally, ethical and regulatory challenges pertinent to AI deployment in clinical practice were thoroughly addressed. A critical “validation gap” often exists between the promising performance reported in initial AI studies and the stringent requirements for widespread clinical adoption and regulatory approval [32, 33]. The current reliance on limited or proprietary datasets, coupled with insufficient external validation, creates a significant barrier to translating AI from research into routine clinical practice. This necessitates a fundamental shift in future research methodologies for AI in CSC, prioritizing large, diverse, multicenter datasets and rigorous, independent external validation studies to build trust in AI models, demonstrate their generalizability, and ultimately achieve broad regulatory acceptance and clinical utility [32–34]. Addressing issues of algorithmic bias, particularly related to demographic factors such as age, sex, and ethnicity, is essential to ensure equitable AI performance across diverse patient populations [35, 36]. The FDA’s total product life cycle approach and emerging regulatory frameworks, including predetermined change control plans for continuously learning algorithms, represent important steps toward safe and effective AI integration in clinical ophthalmology [37, 38]. 

## Results

### AI applications in CSC diagnosis

**Fig. 1:**
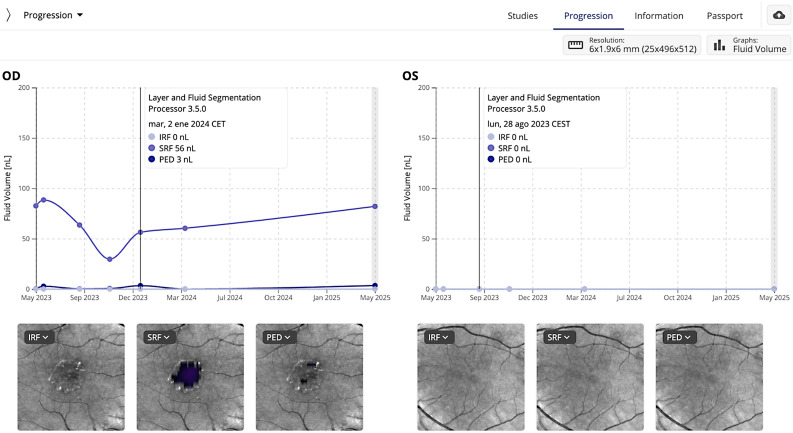
AI-based OCT analysis software for central serous chorioretinopathy: Quantification of subretinal fluid (nL) and other biomarkers (e.g., Retinal Thickness, Atrophy) to monitor treatment response over time

**Fig. 2:**
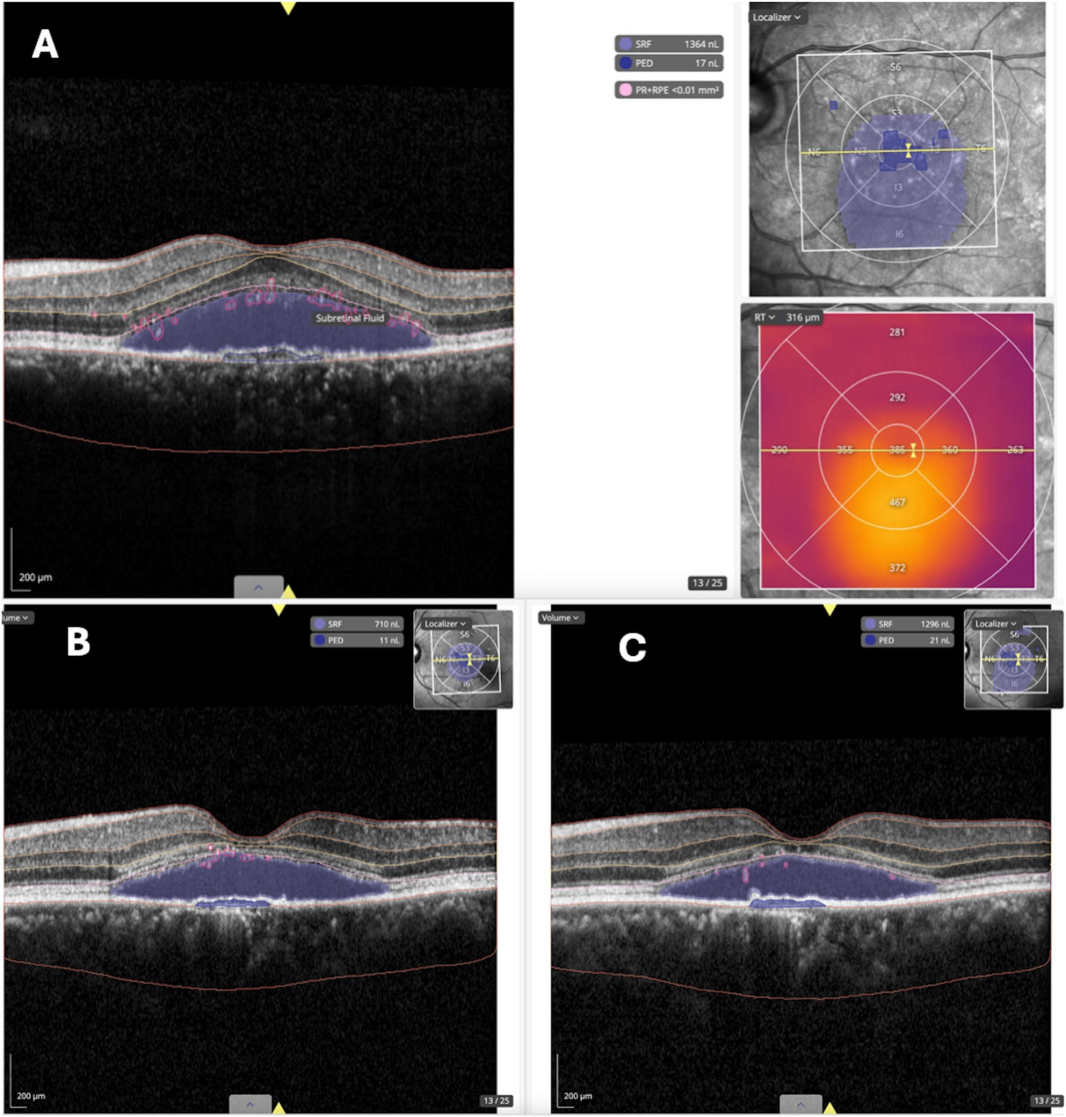
The AI-based OCT analysis software discovery (RetinAI, Bern, Switzerland & Boston, USA) enables (**A**) quantification of subretinal fluid in patients with central serous chorioretinopathy and (**B**, **C**) comparison across multiple visits in the same patient. Although the scans appear visually similar, the follow-up visit (**C**) showed almost double the subretinal fluid (SRF) volume compared with the previous visit three months earlier (**B**)

AI has demonstrated a potentially important impact on CSC diagnosis through advanced capabilities in image analysis and biomarker identification (Figs. 1 and 2). This section details the application of AI across various imaging modalities and its performance in detecting and subtyping CSC (Tables 1 and 2), while emphasizing that diagnostic performance should be interpreted in relation to the datasets, comparator diseases, and clinical context used for model development and validation.

**Table 1:** Scientific applications of artificial intelligence in central serous chorioretinopathy (CSC)

AI application	Scientific basis / methods
Automated diagnosis from multimodal imaging	CNNs trained on OCT, FFA, ICGA and fundus images (Refs. 39,40,58)
Prediction of disease chronicity	Machine learning models based on baseline imaging and clinical features (Refs. 41,47,53)
Subtyping classification (e.g., acute vs. chronic)	Deep learning classifiers using temporal features and choroidal biomarkers (Refs. 50,58)
Response prediction to treatments (e.g., eplerenone, PDT)	Supervised learning on retrospective datasets with treatment outcomes, particularly PDT response (Refs. 52,53,70)
Biomarker extraction from OCT/OCTA	Radiomics features and AI-derived heatmaps, fluid quantification, RPE irregularity, and leakage-related OCT patterns (Refs. 20,46–48)
Differential diagnosis (e.g., CSC vs. AMD or pachychoroid)	Multimodal feature fusion for classification tasks; requires validation against SRF-related differential diagnoses (Refs. 39,40,57)
Monitoring and longitudinal tracking	Time-series and longitudinal modeling of OCT-derived fluid and structural biomarkers (Refs. 20,41,46,47)
Phenotype clustering using unsupervised learning	K-means clustering and hierarchical models on OCT/OCTA datasets (Refs. 50,57,58)
Anterior segment OCT / scleral interface analysis	Automated segmentation of episcleral-scleral interface and anterior scleral thickness for phenotyping and PDT response prediction (Refs. 69,70)

AI, artificial intelligence; CSC, central serous chorioretinopathy; CNN, convolutional neural network; DL, deep learning; ML, machine learning; RF, random forest; LR, logistic regression; SL, supervised learning; UL, unsupervised learning; RNN, recurrent neural network; OCT, optical coherence tomography; OCTA, optical coherence tomography angiography; FFA, fundus fluorescein angiography; ICGA, indocyanine green angiography; AMD, age-related macular degeneration; PDT, photodynamic therapy; RPE, retinal pigment epithelium

This table summarizes the primary research-based applications of artificial intelligence in CSC, including diagnostic automation, disease progression prediction, classification strategies, therapeutic decision support systems, and emerging anterior segment OCT applications. Key references are provided within the table to facilitate access to the primary sources

**Table 2:** Key biomarkers and parameters for AI modeling in central serous chorioretinopathy

Parameter / biomarker	Role
Subretinal fluid height and volume	Diagnostic, quantitative disease burden; longitudinal treatment monitoring (Refs. 20,46–48,53)
Retinal pigment epithelium (RPE) integrity	Predictive of chronicity and treatment response (Refs. 16,53,54)
Hyperreflective foci (HRF)	Marker of disease activity and inflammation (Ref. 44)
Choroidal thickness (CT)	Classification and biomarker of choroidal dysfunction/severity (Refs. 19,45,70)
Choroidal vascularity index (CVI)	Structural marker of choroidal dysfunction (Refs. 19,45)
Double layer sign	Suggestive of chronicity, persistent fluid, and possible neovascular overlap (Refs. 16,53)
OCT-A flow signal alterations	Vascular analysis and ischemia/CNV assessment (Refs. 16,63,64)
Ellipsoid zone (EZ) integrity	Predictive of visual outcome (Refs. 47,53,54)
Presence of pachyvessels	Associated with pachychoroid spectrum (Refs. 7,10)
FFA leakage patterns	Input for automated diagnosis/classification and leakage characterization (Refs. 17,39,40)
ICGA hyperpermeability zones	Correlates with chronic CSC and choroidal hyperpermeability (Refs. 17,21,55)
Symmetry/asymmetry in bilateral cases	Predictive modeling for bilateral involvement and recurrent/persistent disease (Refs. 6,41)
Disease duration	Temporal input in prognostic models and acute/chronic classification (Refs. 3,15,50)
Baseline visual acuity	Prognostic and outcome variable (Refs. 47,53,54)
Anterior scleral thickness / episcleral-scleral interface	Emerging biomarker for CSC phenotype, choroidal outflow hypotheses, and PDT response prediction (Refs. 69,70)

This table details the primary structural and clinical biomarkers used in artificial intelligence models for CSC. These parameters serve diagnostic, prognostic, therapeutic, and classification purposes, particularly in differentiating acute and chronic forms. Key references are included within the table

### Analysis of OCT, OCTA, and FFA images for CSC detection and subtyping

AI models specifically designed for OCT scan analysis have demonstrated high accuracy in detecting CSC and in distinguishing between acute and chronic forms within selected research datasets, with performance often comparable to that of experienced ophthalmologists [39, 40]. A landmark study showed that an AI-based computer-aided diagnosis (AI-CAD) system achieved high detection performance for CSC, with an AUC of 99%, significantly outperforming 66 ophthalmologists in an observer performance test [40]. Crucially, ophthalmologists, including both retina and non-retina specialists, achieved their highest mean diagnostic performance when supported by AI assistance, which included a probability score and a visual evidence heatmap [40]. This augmentation enabled non-retina specialists to approach expert-level diagnostic performance in the tested setting. Nevertheless, these results should not be extrapolated to undifferentiated presentations of SRF without caution. CSC belongs to a broader pachychoroid and retinochoroidal spectrum, and entities such as Vogt-Koyanagi-Harada disease, choroidal tumors, paraneoplastic syndromes, rhegmatogenous or exudative retinal detachment, inflammatory chorioretinopathies, and neovascular macular disease may also present with SRF. Therefore, AI diagnostic systems should currently be considered assistive tools that complement clinical examination and multimodal imaging, rather than standalone substitutes for comprehensive differential diagnosis.

Once a confident clinical diagnosis of CSC has been established, AI in OCT images has shown promising results in predicting disease recurrence and persistence [41]. Beyond OCT, AI applied to FFA images may support faster and more consistent identification of leakage and disease activity. These applications may reduce workload by automating lesion detection, providing diagnostic support, and generating structured outputs, but they require careful validation in datasets that include relevant differential diagnoses and real-world imaging variability.

### AI-assisted biomarker identification

Deep learning (DL) models, particularly convolutional neural networks (CNN), have become the dominant approach for retinal OCT image segmentation [42, 43]. These methods are fundamental for accurately segmenting anatomical structures and pathological lesions, thereby providing crucial biomarkers for the diagnosis of ocular diseases. AI-based OCT biomarker detection has proven effective in identifying specific indicators associated with CSC.

Hyperreflective foci (HF) and flat irregular pigment epithelium detachment (FIPED) were found to be clinically relevant biomarkers, positively associated with CSC at baseline, with HF present in 81% of cases and FIPED in 88–89% across both acute (aCSC) and chronic (cCSC) CSC groups, aiding in diagnosis confirmation [44]. However, despite their utility in diagnosis, these AI-detected HF and FIPED biomarkers alone could not reliably distinguish between aCSC and cCSC at the first visit [44]. While intraretinal fluid (IRF) showed statistically significant differences between the aCSC and cCSC groups, its low overall prevalence (below 50%) suggested limited clinical relevance for consistent differentiation [44]. This indicates that while AI is highly proficient at identifying the presence of CSC and its associated structural abnormalities, its capability to precisely subtype the disease at initial presentation remains an evolving area. The clinical distinction between acute and chronic CSC likely depends on factors beyond the mere presence of static biomarkers, possibly involving temporal changes or more nuanced morphological patterns.

AI-based biomarker segmentation and analysis, applied to FFA images for hyperfluorescence and OCT exams for SRF and length of photoreceptors, provide valuable insights into disease understanding. In choroidal analysis using OCT imaging, AI plays a significant role in the precise detection, quantification, and automated classification of choroidal biomarkers, such as choroidal thickness and vascularity index. AI models are used to segment the choroid layer for thickness measurement and to quantify the choroidal vascularity index and other vessel parameters. Advanced DL models, including modified U-Net, RefineNet, and ChoroidNet, are used to segment choroidal vessels and sublayers in swept-source OCT (SS-OCT) and enhanced depth imaging OCT (EDI-OCT) images, enabling the extraction of key choroidal biomarkers such as volume, vascular index, and density [45]. 

### AI-enhanced SRF segmentation and leakage-pattern characterization

Among the available applications, automated layer and SRF segmentation are particularly compelling in CSC because it directly addresses a clinically relevant, quantifiable endpoint. Manual SRF volume segmentation has been used successfully in research settings, including analyses of SRF morphology and treatment response in chronic CSC, supporting the feasibility of volumetric biomarkers [20]. However, AI-enhanced segmentation offers faster, more reproducible longitudinal quantification of SRF volume, retinal thickness, atrophy, and related biomarkers, and may reduce intergrader variability in both research and clinical workflows [20, 46, 47]. In addition to volumetric assessment, AI-based analysis may enable more precise characterization of the spatial configuration and origin of SRF leakage. Recent work describing outer retinal erosion and outer retinal pinching at the origin of SRF highlights that leakage-related morphology can be captured on OCT and may provide additional structural information beyond total fluid volume alone [48]. These observations are particularly relevant to AI-based platforms such as the RetinAI software illustrated in Figs. 1 and 2, which can support visit-to-visit comparison and quantitative monitoring of SRF behavior.

### Performance metrics of diagnostic AI models

The reported performance of AI models in CSC diagnosis is quantitatively promising. An AI model developed to diagnose CSC using OCT scans demonstrated high performance, achieving an accuracy of 93.8%, a sensitivity of 90.0%, a specificity of 99.1%, and an AUC of 98.9% [39]. While such scores highlight the potential of AI in CSC diagnosis, they should be interpreted with caution because they often reflect performance within specific, possibly limited or highly curated datasets. In particular, single-center studies and models not trained against the full differential diagnosis of SRF may overestimate real-world diagnostic performance. Real-world applicability requires rigorous, independent external validation on diverse patient populations and clinically relevant comparator diseases.

Other models have also shown excellent results. A DenseNet-based architecture applied to OCT images achieved high accuracy in CSC detection. A multilayered support vector machine (SVM) classifier applied to OCT images for the detection of retinal edema, CSC, age-related macular degeneration, and normal cases reported an accuracy of 99.92%, with a sensitivity of 100% and a specificity of 99.86% [49]. A CNN developed for CSC classification reached an AUC of 0.927 and an accuracy of 85.7%. Furthermore, an AI-CAD system achieved an AUC of 0.94, outperforming the average diagnostic performance of human ophthalmologists [40]. Deep learning models have also demonstrated the ability to classify CSC subtypes (acute, non-resolving, inactive, and chronic atrophic) with accuracies ranging from 70% to 76.8% [50]. 

### AI for predicting prognosis, recurrence, and treatment response

AI models have shown considerable promise in predicting the clinical evolution of CSC. These tools can estimate SRF absorption, forecast visual acuity outcomes, and assess the risk of disease recurrence, providing ophthalmologists with valuable data to support individualized prognostic assessments and treatment planning.

### Predictive models for subretinal fluid absorption and visual acuity outcomes

Machine learning (ML) algorithms have shown strong potential in predicting SRF absorption in CSC patients following laser treatment, particularly for long-term outcomes. In internal validation, the Random Forest algorithm exhibited superior performance for SRF absorption prediction, with accuracies of 0.651 ± 0.068 at 1 month, 0.753 ± 0.065 at 3 months, and 0.818 ± 0.058 at 6 months. For external validation, the best performance was observed with the XGBoost algorithm, reaching accuracies of 0.734 at 1 month, 0.727 at 3 months, and 0.900 at 6 months. Across all models, SRF absorption prediction at 6 months post-treatment consistently yielded the highest accuracy, while short-term predictions (within three months) were relatively less precise [51]. 

**Fig. 3:**
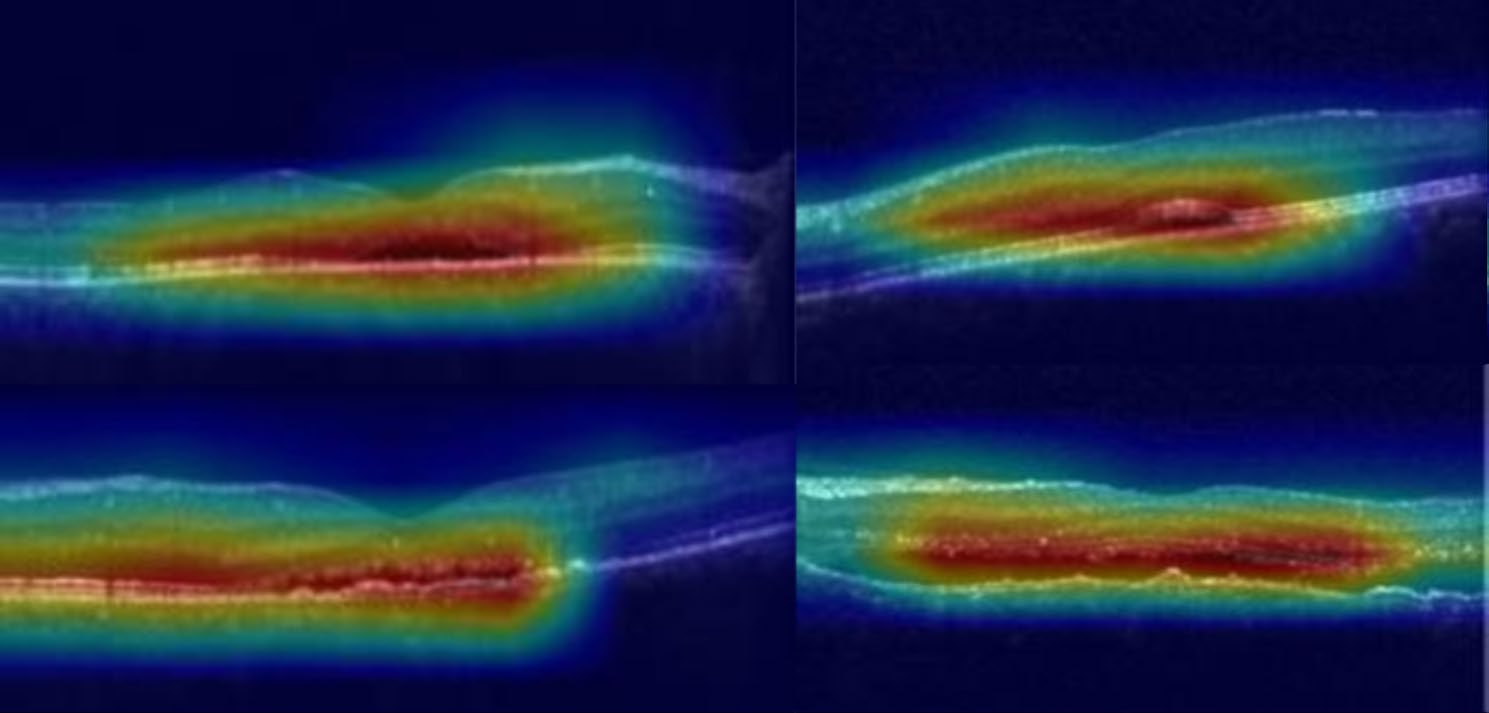
OCT images from different patients with central serous chorioretinopathy (CSC) analyzed using AI-based image analysis. The class activation maps, generated using the Grad-CAM algorithm and based on the DenseNet-121 architecture, highlight the most relevant retinal regions contributing to the model’s prediction. A color scale ranging from 0 (cold colors) to 1 (warm colors) reflects the level of confidence in each region’s importance. Areas with subretinal fluid appear in red, indicating a greater influence on the final prediction. These maps provide insight into the neural network’s internal decision-making and enhance the interpretability of the AI model’s output

One of the earliest studies to use DL to predict treatment response in chronic CSC developed and validated a CNN trained on baseline spectral-domain OCT (SD-OCT) B-scans to predict anatomical response to half-fluence photodynamic therapy (PDT) at 3 months [52]. A total of 216 eyes were classified into three outcome categories: complete, partial, or no resolution of SRF (Fig. 3). The model used a ResNet-50 backbone with transfer learning and data augmentation to improve generalizability and reduce overfitting. Among the classification approaches, binary separation between complete responders and partial/non-responders achieved an accuracy of approximately 67%, while differentiating between partial and non-responders produced precision values of 0.74 and 0.69, respectively. Despite the inherent heterogeneity of CSC, the model demonstrated the feasibility of using OCT-based AI to deliver clinically relevant, image-driven predictions before treatment. These results support the use of AI in personalized treatment planning, especially for PDT.

AI has also been used to predict post-therapeutic visual acuity (VA) and OCT images in patients with CSC after laser treatment, representing a novel application in the field [53]. For VA predictions, a blending algorithm demonstrated the highest accuracy, with mean absolute errors (MAEs) of 0.074 logMAR at both 1 and 3 months, and 0.091 logMAR at 6 months. Root mean square errors (RMSEs) were 0.096 logMAR (1 month), 0.103 logMAR (3 months), and 0.119 logMAR (6 months) [53]. For anatomical prediction, a pix2pixHD model based on a generative adversarial network architecture was used to generate synthetic OCT images after treatment. Synthetic OCT images generated by this method were largely indistinguishable from real images, with 95.31% identified as real at 1 month and 93.94% at 3 months. MAEs of central macular thickness (CMT) for synthetic OCT images were 30.15 ± 13.28 μm for 1-month predictions and 22.46 ± 9.71 μm for 3-month predictions [53]. This capability to predict visual and anatomical outcomes can significantly alleviate emotional stress for patients and provide ophthalmologists with objective references for treatment selection.

### AI in identifying prognostic factors for CSC

ML models have proven valuable in identifying key features that significantly impact SRF absorption and, consequently, the prognosis of CSC patients. Beyond the specific laser therapies, these factors include SRF height, central macular thickness (CMT), double-layer sign (DLS), and even patient-reported scores on the Pittsburgh Sleep Quality Index and the Hamilton Anxiety Scale. These findings underscore the relevance of combining clinical imaging features with lifestyle and psychological factors to develop comprehensive prognostic assessments.

Baseline retinal features, such as ellipsoid zone (EZ) integrity, retinal neuroepithelial layer (RNEL), and CMT, have been identified as critical predictors of VA prognosis [53, 54]. Choroidal thickness has been recognized as a significant predictor of treatment response in CSC, influencing the choice between therapies such as eplerenone and PDT.

AI, particularly through analysis of fundus and OCT images, has shown promising capabilities in predicting disease recurrence in CSC. A ResNet50 model using B-scans and en face images derived from retinal thickness achieved accuracies of 0.8072 and 0.92, respectively, in predicting persistent CSC [41]. Studies further suggest that patients with complex CSC, characterized by bilateral involvement or a history of previous episodes, face a higher risk of SRF recurrence and may benefit from early interventions rather than observation. These capabilities support the development of personalized management strategies and optimized follow-up schedules.

### AI-guided treatment strategies for CSC

The application of AI to guide treatment strategies for CSC is an emerging field focused on personalizing and optimizing therapeutic interventions. Although fully autonomous AI-guided treatment protocols remain in early development, AI is increasingly contributing to clinical decision support and outcome prediction, laying the groundwork for more individualized and optimized disease management.

PDT has been a cornerstone in the treatment of chronic CSC, demonstrating short-term efficacy [55, 56]. While PDT has shown favorable clinical outcomes, its application can be challenging due to factors such as the worldwide shortage of verteporfin, the photosensitizer used in PDT. AI’s role here is to identify optimal candidates for PDT by predicting treatment response using multimodal imaging biomarkers, such as choroidal thickness. This could help manage resource scarcity and improve patient selection for a therapy that creates free oxygen radicals to induce choroidal vascular remodeling, reducing hyperpermeability and mitigating SRF leakage.

AI’s ability to analyze large datasets, including patient demographics, medical history, genetic information, and imaging data, offers a pathway to personalized treatment plans. By predicting individual responses to therapies, AI can optimize patient outcomes and potentially reduce healthcare costs. For instance, AI-driven models can identify which patients are likely to respond to specific treatments, enabling more targeted interventions. This approach does not replace the established role of PDT in chronic CSC, but rather may help refine patient selection, timing of intervention, retreatment decisions, and follow-up strategies within evidence-based care pathways.

Recent efforts have focused on developing individualized management strategies based on predictions of SRF absorption derived from big data, with ML models identifying key features relevant to CSC patients’ prognoses, including SRF height, CMT, DLS, and even sleep quality and anxiety scores. Additionally, AI tools have been used to predict post-treatment VA across different therapeutic options, further aiding the selection of the most appropriate and cost-effective interventions [53]. 

### Role of AI in monitoring disease progression and complications of CSC

AI plays an increasingly important role in the continuous monitoring of CSC and in detecting potential complications. Its capacity for automated, precise, and longitudinal analysis of ophthalmic imaging enables improved assessment of disease progression and therapeutic response [46, 57]. 

AI-based algorithms are increasingly used to monitor retinal diseases, including CSC, particularly by analyzing fundus and structural OCT images [58]. OCT is a critical tool for monitoring chronic CSC patients and tracking SRF resolution. AI’s ability to accurately segment layers and calculate volumes/areas from OCT images makes it effective for detecting even small amounts of fluid, quantifying longitudinal SRF changes, and characterizing spatial fluid patterns [20, 46–48]. This precision is vital for assessing anatomical response to therapy, determining the need for retreatment, and standardizing endpoints across clinical studies.

Beyond fluid detection, AI-enabled biomarker analysis deepens understanding of CSC-related complications [57]. In particular, the long-term presence of SRF in CSC can lead to elongation of photoreceptor outer segments, a change that AI algorithms can automatically detect and analyze across large patient cohorts. Studies have shown that both foveal SRF and elongated photoreceptors are significantly associated with reduced VA, whereas other factors, such as hyperfluorescence or SRF height, may be less predictive of VA deterioration [59–61]. Importantly, this photoreceptor elongation can reverse after fluid resorption, without leaving permanent effects on final VA [59]. These AI-enabled insights highlight photoreceptor integrity as a key determinant of visual function and provide a more nuanced understanding of the relationship between anatomical changes and functional outcomes in CSC [47, 62]. 

Beyond fluid and photoreceptor changes, AI image interpretation systems show promise in predicting CSC complications, including choroidal neovascularization (CNV) [57, 63, 64]. AI can help clinicians identify early pathological signs of CNV or concurrent conditions more quickly, enabling earlier intervention and improved patient outcomes. Its precision in layer segmentation and spatial quantification makes AI particularly valuable for detecting persistent or recurrent CSC, where anatomical changes may be subtle or overlooked in routine evaluation [58]. DL algorithms, in particular, are adept at identifying complex patterns without explicit programming, making them essential tools for monitoring progression and detecting CSC complications.

### Comparative analysis: AI-based approaches versus traditional diagnostic and prognostic methods in CSC

The integration of AI into ophthalmology has prompted direct comparisons with traditional diagnostic and prognostic methods for CSC. AI-based systems offer potential advantages in accuracy, processing speed, and objectivity, challenging the traditional reliance on manual image interpretation and clinician experience.

### Diagnostic accuracy and efficiency

Traditional diagnostic approaches to CSC rely on physician expertise and multimodal interpretation. In contrast, AI-based systems have demonstrated high diagnostic accuracy in controlled datasets and may improve consistency and efficiency. For example, an AI-CAD system for CSC achieved an AUC of 0.99, outperforming a cohort of 66 ophthalmologists in an observer performance test [40]. When supported by AI, including probability scores and visual evidence heatmaps, ophthalmologists showed a marked improvement in diagnostic accuracy. The average AUC improved from 0.901 to 0.941 for all ophthalmologists, from 0.878 to 0.922 among non-retina specialists, and from 0.921 to 0.956 for retina specialists [40]. This demonstrates that AI can act as an “expert amplifier” in appropriate clinical contexts. However, because many CSC models have not been trained on a broad range of SRF-related differential diagnoses, such systems should be used as clinical decision-support tools rather than as definitive, autonomous diagnostic systems.

In addition to improving accuracy, AI substantially enhances diagnostic efficiency. Automated analysis of FFA images has been shown to significantly reduce diagnostic time while maintaining or improving accuracy, especially for large or complex datasets. By automating routine diagnostic tasks, AI systems reduce the burden on ophthalmologists, allowing them to focus on clinical decision-making and more complex patient care.

### Prognostic capabilities in CSC

Prognosis in CSC has traditionally depended on clinical judgment and subjective parameters, such as symptom duration and fundus appearance, which often fail to capture the complexity of chronic or recurrent cases. While acute CSC frequently resolves without intervention, chronic forms can lead to persistent SRF, outer retinal atrophy, and irreversible vision loss [1, 2]. 

AI-based prognostic models offer a more objective and quantifiable alternative. ML algorithms, such as XGBoost, have demonstrated high accuracy in predicting SRF absorption with reported accuracies of up to 0.900 at 6 months. Additionally, these models can forecast post-treatment VA and simulate future OCT B-scans, offering predictive insights up to six months in advance [53]. This capability is especially valuable for identifying patients at risk of chronic CSC and guiding timely therapeutic decisions.

AI also helps uncover key prognostic biomarkers directly relevant to CSC. Variables such as SRF height, CMT, DLS, and even non-ocular factors like patient anxiety have been identified as significant predictors of disease progression and persistence. The ability of AI to integrate structural OCT parameters and systemic risk factors enables a stratified risk assessment and supports personalized follow-up regimens. Furthermore, DL models trained on longitudinal imaging data can detect subtle signs predictive of recurrence, which are often overlooked in conventional evaluations [41]. 

By replacing subjective estimates with data-driven predictions, AI enhances the precision of prognostication in CSC, optimizes resource allocation, and supports early intervention strategies for patients at risk of chronicity or vision loss.

## Challenges, limitations, and ethical considerations of AI implementation in CSC

Despite its potential, integrating AI into CSC management faces important technical, regulatory, and ethical challenges. Many models perform well on internal datasets but lack robust external validation, limiting generalizability—particularly in chronic CSC populations [32, 33]. Restricted demographic diversity, reliance on proprietary data, and limited reproducibility further compromise reliability and transparency [34, 35]. 

Regulatory standards also vary: while many CE-marked AI devices rely primarily on retrospective data, the FDA increasingly requires prospective, multicenter validation [37, 65]. Notably, a substantial proportion of ophthalmic AI tools lack strong peer-reviewed evidence, underscoring the need for higher-quality clinical validation and real-world data [65, 66]. 

In addition, the “black-box” nature of many deep learning systems limits interpretability and may hinder clinical trust [22, 67]. Enhancing explainability and aligning outputs with clinical reasoning are essential for adoption. Finally, privacy and equity remain critical concerns: ocular imaging data may be re-identifiable, requiring advanced privacy-preserving strategies, and AI systems trained on non-representative populations risk perpetuating healthcare disparities [35, 36, 68]. Robust validation, transparency, and inclusive dataset design are therefore fundamental to safe and equitable AI deployment in CSC.

## Future directions and emerging trends for AI in CSC research and clinical practice

The future of AI in CSC lies in its transition from isolated diagnostic algorithms to fully integrated, multimodal, and clinically deployable systems that support precision medicine.

A key priority is multimodal data integration, combining structural and vascular imaging (OCT, OCTA, FFA, FAF) with longitudinal clinical data, treatment history, and potentially genetic information. Such integrative models may improve disease subtyping, biomarker discovery, and therapeutic response prediction, moving beyond single-modality analysis toward comprehensive disease modeling. Achieving this will require standardized data curation, interoperable infrastructures, and close collaboration between ophthalmology, bioinformatics, and data science.

AI is also poised to expand within teleophthalmology and remote monitoring, particularly for chronic or recurrent CSC. Automated analysis of home- or technician-acquired OCT images could enable early detection of SRF recurrence and support individualized retreatment timing, reducing the burden of in-person visits while maintaining specialist-level oversight. Successful implementation will depend on CSC-adapted imaging protocols and structured digital referral pathways.

Another promising, still understudied direction is extending AI to anterior segment OCT in CSC. Existing AI models are better developed for posterior pole layer and fluid segmentation, but analogous methods could be trained to delineate the episcleral-scleral interface and quantify anterior scleral thickness. This is relevant because swept-source OCT studies have shown increased anterior scleral thickness and visible supraciliary space in CSC eyes, suggesting a potential relationship between scleral anatomy, choroidal outflow, and pachychoroid-related disease [69]. More recently, anterior scleral thickness has been associated with complex CSC phenotypes and with response to half-dose PDT, suggesting potential therapeutic implications [70]. Automated anterior-segment OCT segmentation could therefore provide additional biomarkers for patient stratification, treatment-response prediction, and mechanistic studies of choroidal venous congestion.

In parallel, AI-driven transformation of large imaging repositories into structured real-world evidence (RWE) offers a powerful tool for understanding long-term treatment response and disease heterogeneity. By harmonizing multicenter datasets, AI can facilitate the identification of prognostic biomarkers, optimize therapeutic algorithms (e.g., for PDT or mineralocorticoid receptor antagonists), and complement conventional clinical trials—an approach increasingly recognized by regulatory agencies [37, 71]. 

Ultimately, these advances converge toward personalized and precision medicine, where AI-guided models integrate multimodal and longitudinal data to deliver individualized diagnostic and therapeutic strategies. In a disease as heterogeneous and variably recurrent as CSC, such data-driven personalization represents one of the most promising avenues for improving long-term outcomes.

## Conclusion

AI is rapidly influencing the landscape of CSC management, offering important advances in diagnosis support, prognosis, and treatment planning. In particular, AI’s integration with imaging modalities such as OCT has achieved high diagnostic accuracy in selected datasets, while simultaneously improving workflow efficiency through automation and decision support. However, diagnostic claims should be interpreted cautiously until models are validated in diverse populations and against clinically relevant SRF-related differential diagnoses. AI models are now capable of detecting key CSC biomarkers, such as hyperreflective foci, flat irregular pigment epithelial detachment, SRF volume, and leakage-related OCT patterns, and are increasingly accurate at predicting SRF absorption, VA outcomes, and recurrence risk. These capabilities support a more personalized approach to disease monitoring and follow-up scheduling.

Although AI-guided treatment strategies for CSC are still under development, early applications show promise for predicting treatment responses to PDT and laser-based interventions, as well as for optimizing longitudinal monitoring. This contributes to better resource allocation and individualized therapy selection. Furthermore, the incorporation of AI into clinical workflows may enhance diagnostic performance across different levels of expertise, especially benefiting general ophthalmologists and reducing clinical workload when used as an adjunct to comprehensive clinical assessment.

Nonetheless, several challenges remain before AI can be fully integrated into routine CSC care. These include the need for external validation in diverse populations, mitigating dataset bias, and harmonizing regulatory requirements across regions. In addition, the “black-box” nature of some algorithms poses interpretability issues, and ethical considerations related to data privacy, transparency, and equitable access must be carefully addressed.

Looking forward, the integration of multimodal data, including imaging, genetic, clinical, and anterior segment OCT-derived scleral information, will be key to advancing precision medicine in CSC. AI-enabled teleophthalmology and remote monitoring offer promising avenues to expand access to specialist care in underserved areas. At the same time, the growing regulatory acceptance of RWE generated through AI may accelerate clinical adoption and therapeutic innovation.

To fully realize the potential of AI in CSC, ongoing collaboration among clinicians, researchers, ethicists, and policymakers will be essential. Transparent algorithm development, rigorous validation, and a commitment to equity will help ensure that AI not only enhances patient outcomes but also contributes meaningfully to the evolution of ophthalmic care.

## Data Availability

No datasets were generated or analysed during the current study.
